# Age and gender in gerontechnology development: Emphasizing the need for an intersectional approach

**DOI:** 10.1007/s00391-023-02183-2

**Published:** 2023-05-11

**Authors:** Katja Antonia Rießenberger, Florian Fischer

**Affiliations:** Bavarian Research Center for Digital Health and Social Care, Kempten University of Applied Sciences, Albert-Einstein-Straße 6, 87437 Kempten (Allgäu), Germany

**Keywords:** Participation, Digital divide, Paternalism, Co-creation, Co-construction, Partizipation, Digitale Kluft, Paternalismus, Ko-Kreation, Ko-Konstruktion

## Abstract

**Background:**

The challenges of the digital divide emerge with new technologies being created to address the needs of the increasing older population. This divide is influenced by the social dimensions of age and gender, often resulting in impaired participation of the affected demographic groups. Gerontechnological designs in which inadequate attention is paid to gender and old age easily run the risk of reproducing gender-specific and age-specific stereotypes. An approach to counteracting the digital exclusion of technology users is the introduction of co-creative methods of participatory design (PD). As there are diverse challenges when putting these methods into practice regarding their claim to be more socially inclusive and democratizing technology development, it is necessary to investigate the effect that age and gender could play when considering PD in gerontechnology.

**Objective:**

This article aims to shed light on the intersection of age and gender as dimensions of horizontal inequalities in gerontechnology development to support the further development of co-creation practices.

**Conclusion:**

The PD approaches can be regarded as suitable methodologies to descript age and gender in technology development as long as they are enhanced by a critical awareness of gendered and ageist patterns in society and technology development. The intersectional approach can contribute to further understanding of how current gerontechnology development practices promote the reinforcement and challenging of dominant discourses on old age and gender.

## Abstract (de)

**Hintergrund:**

Die Herausforderung der digitalen Kluft kann durch die Entwicklung neuer Technologien zur Deckung des Bedarfs der wachsenden älteren Bevölkerung verstärkt werden. Diese Kluft wird durch die sozialen Dimensionen Alter und Geschlecht beeinflusst und führt häufig zu einer eingeschränkten Teilhabe der betroffenen demografischen Gruppen. Gerontotechnologien, bei denen Gender und Alter nicht adäquat berücksichtigt werden, laufen leicht Gefahr, gender- und altersspezifische Stereotype zu bedienen. Ein Ansatz, um der digitalen Exklusion von Technologienutzer:innen entgegenzuwirken, ist die Einführung kokreativer Methoden partizipativen Designs (PD). Da die Umsetzung dieser Methoden hinsichtlich ihres Anspruchs, sozial inklusiver zu sein und die Technologieentwicklung zu demokratisieren, mit diversen Herausforderungen verbunden ist, besteht die Notwendigkeit, den Einfluss von Alter und Gender bei der Betrachtung des PD in der Gerontotechnologie zu untersuchen.

**Ziel der Arbeit:**

Der Beitrag soll die Intersektionalität von Alter und Gender als Dimensionen horizontaler Ungleichheiten in der Gerontotechnologieentwicklung beleuchten, um die Weiterentwicklung von Kokreationspraktiken zu fördern.

**Schlussfolgerung:**

PD-Ansätze können als geeignete Methoden zur Erschließung von Alter und Gender in der Technologieentwicklung angesehen werden, sofern sie durch ein kritisches Bewusstsein für gender- und altersspezifische Muster in der Gesellschaft sowie bei der Entwicklung von Technologien ergänzt werden. Der intersektionale Ansatz kann dazu beitragen, ein besseres Verständnis dafür zu entwickeln, wie die gegenwärtigen Praktiken der Gerontotechnologischen Entwicklung die vorherrschenden Diskurse über das Alter und Gender verstärken oder in Frage stellen können.

## Introduction

While new technologies are being created to address the needs of the rising older population in all industrialized countries [25], the challenge of the digital divide further emerges. This divide is generally distinguished by a social disintegration deriving from an unequal distribution of access to, usage of, and benefits of technology. As it is largely influenced by the social dimensions of age and gender, it often results in impaired participation of the affected demographic groups [5, 8]. This paper aims to shed light on the intersection of age and gender as dimensions of horizontal inequalities in gerontechnology development to support the further implementation of participatory design practices.

## Social construction of age(ing) and gender

Leontowitsch and Werny described two major positions in society and science on the social construction of age(ing) and gender, with one position being that both age(ing) and gender are attributed to biological and physiological processes. They are thus seen as inherently different categories, which seemingly confirms a “natural dichotomy between male and female as well as old and young” (p. 2) [17].

In diametric opposition, the second position is West and Zimmerman’s constructivist doing approach [30]. It assumes that the category of gender is not naturally given and instead a social construct offering a certain spectrum for its formation [17]; however, despite the fact that age(ing) is also considered a processual category because everyone experiences the various stages of aging, it is still a result of social attributions and discourses and thus a social construction, similar to gender [17]. Still, “age” is hardly acknowledged or included as a social construction in gender research [11, 17]. Furthermore, comprehensive research on the ageing of men and women is only possible if ageing and gender are viewed as mutually operating sociostructural and socioprocedural categories and constructs in their impact over the life course [1, 17].

Following an intersectional perspective on these social constructions of age and gender, it is assumed that simply adding categories emphasizes differences, which presupposes an ideal in society and technology development. In the case of old age and gender, this is the ideal of the old man to whom old women are compared [16, 17, 27].

In the advanced stage of old age, gender-specific distinctions seem to become less integral with dependency and diminishing agency becoming more apparent due to severe physical decline and need for care. Nonetheless, even in advanced old age, older women are still affected by age-related negative associations and discrimination to a greater extent than older men [17]. Advanced age in combination with gender may have a negative influence on older women, with serious repercussions for their health and well-being. Such gendered ageism often renders older women largely invisible and is embedded in contemporary culture and social and economic policies [17, 26].

## Old age in technology development

Both design paternalism and age scripting have been described in the context of co-creation and gerontechnology development. According to Khadilkar and Jagtap [14], paternalism requires the presence of three factors: “1) a decision maker (the paternalist) making a decision that interferes with the autonomy of the target of the decision, 2) the decision is made without the consent of the target and 3) the paternalist believes that the decision will lead to beneficial effects for that target group”. In the context of design paternalism and older age, it describes the often implicit assumption that older adults should not be disturbed by technologies in their everyday life or when using them. They are thus at times denied creative appropriation processes of technologies as well as their autonomy and freedom in using said technologies [22].

Age scripting, on the other hand, describes a process that is based on the premise that age(ing), technologies, and social contexts are inextricably linked. The age discourses based on this are then inscribed unfiltered into new technologies during the process of technology development [28], which in turn influences technology development unreflectively and therefore often unnoticed.

Design paternalism and the process of age scripting are interdependent in so far as design paternalism can be seen both as a cause of age scripting (as part of age discourses) and as a result of it (based on design paternalism emerging through age scripts). These age scripts as well as design paternalism are described as a cause of ageism in the field of science and technology studies (STS) and gerontechnology because of their restrictive, exclusionary, and possibly also disempowering nature [23].

## Gender in technology development

Gender has long been considered an important factor in shaping participation in technology development. Yet, even though studies have identified women as more active users of digital technologies than men, there is still a gender divide in terms of uneven access to information and communication technologies (ICT), skills, and infrastructure [12]. Studies suggest that gender often plays a role in this with certain user groups needing to make a greater effort than others to access technology or to make it meaningful to them [21]. Designs in which no particular attention is paid to gender easily run the risk of being gender-specific and therefore more directed toward men [29]. Parallel with age scripts, this approach in technology development becomes even more problematic when gender stereotypes are inscribed into the technology (gender scripts). For example, when the design reflects the assumption that women are technologically incompetent [10], even when the contrary seems to be true: women constitute a diverse and demanding user base, with the ability to bring new angles to topics like ICT applications [5].

## Participatory design and co-creation

In order to be able to change the design of technologies and apply more appropriate design methods, developers of technologies need to understand in which sense their products might be problematic in age and gender issues. One approach to counteract the digital exclusion of technology users is the introduction of co-creation workshops, as a method of participatory design. It is an attempt in innovation processes of digital technologies to democratically involve and therefore increase the chance of digital inclusion of groups, such as people aged 65+ years whose heterogeneities are systematically disregarded and whose potential vulnerabilities are overused [3].

In participatory design it is essential to involve the people whose lives will be changed by the results of a process. Although this is the case, little critical thought is given to how people are involved, their roles in projects, and how their involvement is managed over time [15]. Additionally, recent research shows that even in these co-creation processes, sexist and ageist elements, e.g., design paternalism, age scripting, or the previously mentioned gender scripts, still come into play [10, 23]. Furthermore, participants are too often defined as homogeneous entities and addressed in a simplistic manner, which neglects intersubjective nuances, including aspects such as gender, age, education, ethnicity, and socioeconomic status [19]. Even when including older adults in the process of technology development, it needs to be considered that older persons are always going to be diverse. Differences in social status, economic status, gender, personal history, or technology literacy can have a major impact on how technology is or is not appropriated, how it is being used, or how it is being modified by the end user.

In the same way, as individual life worlds can therefore influence technology development and usage, images of ageing and gender can do the same. Age and gender studies have long shown that public images of ageing and gender perpetuated in the media or marketing practice, shape the cultural lens through which we perceive and evaluate age(ing) and gender. Although yet understudied, it can be assumed that these views on ageing and gender (of individuals and in broader society) are crucially linked with the development of technologies. Images of ageing and gender play an integral and pivotal part that influence technology designs and how these technologies are marketed. Societally and personally held stereotypical views can reach an ageist and sexist level and still end up getting implemented in technology design. This can, e.g., be traced back to the often subconscious nature of said stereotypes and a lack of awareness of such biases. These images then not only perpetuate the public image of old age and gender but also beliefs that, e.g., older adults incorporate about themselves and may end in self-ageism and internalized sexism [2, 24]. This pattern has been thoroughly observed and questioned by different researchers [13, 24].

Another main challenge that comes into play, even during participatory design processes, is when technology development is based on an interventionist logic. Here, technology developers and social scientists approach aging as a “target for interventions or as a set of problems to be solved” (p. 4) and older adults are perceived as in need of technological interventions and an implicit but strong relation between ageing and care is implied [24]. This again in turn draws back to a mindset also found in design paternalism, where decisions are being made on behalf of another person or entity without their knowledge and consent and yet justified by the claim that they will be protected from harm or even that it will enhance their lives [14].

Consequently, when considering participatory design practices, such as co-creation as an approach to gerontechnology development, it is important to investigate the effect that age and gender might play, especially in a group setting [5], with gender being one of the different identities “negotiated both through personal interactions and in the materials used and objects created during the participatory design processes” [4, p. 2]. Oudshoorn et al. [20] described that even though they had used participatory and user-centered design strategies, the feedback provided was interpreted in a very selective manner and at large not considered. Nonetheless, as strategies that aim at narrowing the digital divide in terms of gender and age(ing) work against traditional stereotypes of gender and age(ing), participatory design approaches can be regarded as suitable methodologies to describe age and gender in technology development, as long as they are enhanced by a critical awareness of gendered and sexist as well as ageist patterns in society and more specifically in technology development [10, 24]. With the emphasis on the situatedness in which these projects now emerge and the different types of participants, new demands are placed on such project reflection [15].

## Intersectionality in technology development

Based on Crenshaw [9] who assumed that culturally constructed categories, such as gender, class, race, and age interact on multiple levels, it is important to understand the subject construction of and connection between the categories of age and gender. As two intersecting dimensions in gerontechnology development, the methodology of intersectionality appears to be suitable for this purpose.

At the intersection between age studies and science and technology studies (STS), the field of gerontechnology slowly unfolds its potential scope and has begun to renew naïve techno-deterministic views of ageing and technology owing to the increase of empirical research on the design and usage of technology by and for older adults [23]. Due to the heterogeneity of old age and ageing, the intersectional approach can contribute to further understanding which technology may both reinforce and challenge prevailing images of older adults. [20].

Nonetheless, the question of how diversity of older adults relates to technology design has so far remained untouched, in contrast to gender and technology. It became clear that there is sufficient content regarding gender aspects and technology innovation and development [10, 12]; however, a critical reflection of this research area showed that most studies on gender and technology innovation and development remain unaware of other sociocultural categories of diversity including class, ethnicity, disability, sexuality, and age [18, 20]. Even though “gender and age are important, co-constructed, and structural systems of inequality” (p. 3) [6], the intersection between the two has been particularly neglected among scholars and in intersectionality theory [7]. There are very few studies reflecting on age(ing) and participatory design of technologies on a metalevel and close to none when it comes to the intersection of age and gender in participatory design processes and technology development. While Oudshoorn et al. [20] focused on age and gender in technology development, the aspect of gender was restricted to young girls. While Buchmüller et al. [5] tried to combine the aspects of age, gender, and technology development, they only included study participants up until the age of 65 years, fully excluding adults aged 65 years and older.

As a hitherto understudied area of intersectional analysis at the junction of age, gender, and technology, it is recommended to follow Joyce and Mamo’s [13] encouragement to study said intersection. A critical reflection of (1) images of ageing and gender in gerontechnology development and (2) how participatory design processes are enacted is needed as well as (3) what and how related age and gender dimensions are performed by actors to illuminate the intersection of age, gender, and gerontechnology in participatory design processes, such as co-creation. For future studies and gerontechnology development, it is furthermore recommended to reflect on whether and how age and gender stereotypes can be made useful without overusing them and therefore reproducing ageist and sexist notions in gerontechnology.

## Practical conclusion

- The digital divide is distinguished by a social disintegration deriving from an unequal distribution of access to, usage of, and benefits of technology.
- Gerontechnology designs in which no adequate attention is paid to gender or old age easily run the risk of reproducing gender-specific and age-specific stereotypes.
- It is necessary to investigate the effect of age and gender in gerontechnology development as dimensions of horizontal inequalities.
- An approach to counteracting the digital exclusion of technology users is the introduction of co-creative methods of PD.
- The intersectional approach can contribute to further understanding how current gerontechnology development practices promote the reinforcement and challenging of dominant discourses on old age and gender.
- PD approaches need to be enhanced by a critical awareness of gendered and ageist notions in society at large and more specifically in digital technology development to be regarded as suitable to descript age and gender in technology development.

## Declarations

For this article no studies with human participants or animals were performed by any of the authors. All studies mentioned were in accordance with the ethical standards indicated in each case.
